# Sleep Impairment in Oncology: Development of a Novel Patient-Centric Conceptual Model

**DOI:** 10.3390/cancers18193191

**Published:** 2026-10-02

**Authors:** Harriet Makin, Sophie Van Tomme, Natalie V. J. Aldhouse, Samantha Wratten, Helen Kitchen, Chloe Carmichael, Cecile Gousset, Paul Cordero

**Affiliations:** 1 Clinical Outcomes Assessment, Clarivate Analytics, 70. St Mary Axe, London EC3A 8BE, UK; harriet.makin@clarivate.com (H.M.); natalie.aldhouse@nottingham.ac.uk (N.V.J.A.); sam.wratten@iqvia.com (S.W.); helen.kitchen@clarivate.com (H.K.); chloe.carmichael@clarivate.com (C.C.); 2 Patient-Driven Medicines Development, Sanofi, Paasheuvelweg 25, 1105BP Amsterdam, The Netherlands; sophie.vantomme@sanofi.com; 3 Patient-Driven Medicines Development, Sanofi, 82 Avenue Raspail, 94255 Gentilly, France; cecile.gousset@sanofi.com; 4 Patient-Driven Medicines Development, Sanofi, 410 Thames Valley Park Dr, Earley, Reading RG6 1PT, UK

**Keywords:** cancer, sleep, patient experience, cancer-related sleep impairment, conceptual model, qualitative research, health-related quality of life

## Abstract

Background/Objectives: Up to 95% of cancer patients report sleep disturbances or disorders. These can potentially influence cancer development and outcomes. This study aimed to review the existing literature to understand the patient experience of cancer-related sleep impairment across indications and treatments, to improve awareness and inform disease treatment/management. Methods: A targeted literature review of published qualitative studies exploring sleep impairment in oncology was conducted in MEDLINE, Embase, and PsycINFO. Concepts were extracted from the reviewed articles, and patient quotes or author descriptions/interpretations were subject to secondary analysis using semantic, qualitative, directed content analysis techniques via ATLAS.ti. Results informed the development of a patient-centric conceptual model. Results: From 482 identified publications, 22 publications were selected for in-depth review and concept extraction, collectively including 531 patients across 25 different oncology indications. Cancer-related sleep impairment frequently affected patients living with cancer, who reported difficulty obtaining good quality sleep, including difficulty falling asleep and staying asleep. Sleep problems were triggered by the emotional anxiety of life with cancer, cancer-related symptoms, treatments and side effects, and external factors. Poor sleep greatly impacted daily life for patients, with difficulties in daily life causing further sleep impairment, in a cyclical relationship. Conclusions: This study determined that there is an unmet need for treatment of sleep impairment in oncology to improve patient outcomes. The holistic conceptual model developed in this study can provide an important basis for selecting, developing, or modifying outcome measures to assess relevant sleep outcomes, facilitating discussion between patients and clinicians and improving disease treatment/management.

## Simple Summary

Disrupted sleep has the potential to facilitate cancer development and progression, and most cancer patients report sleep disturbances or disorders. This study aimed to review the existing literature to comprehensively understand the patient experience of cancer-related sleep impairment across indications and treatments. Cancer-related sleep impairment frequently has a considerable impact on daily life for patients across cancer indications and disease stages globally, which in turn causes further sleep impairment, in a cyclical relationship. The conceptual model developed using the insights from the literature review can provide an important basis for selecting, developing, or modifying outcome measures to assess relevant sleep outcomes in oncology patients, facilitating discussion between patients and clinicians and improving disease treatment/management.

## 1. Introduction

Up to 95% of cancer patients report sleep disturbances or disorders during diagnosis, treatment, or after 10 years of survivorship [1]. Severe sleep difficulties, including insomnia symptoms or syndromes, are experienced by 30–50% of cancer patients [1]. The causes of sleep impairment are varied and may include physical changes that are associated with the cancer, the side effects of treatment, stress or worry, and pain or other symptoms related to the cancer [2]. However, there may also be a bidirectional relationship between sleep and cancer, with studies reporting that patients with cancer frequently experience sleep disorders prior to beginning treatment with radiotherapy or chemotherapy [3]. As long-term sleep disruptions may raise the risk of some cancers [4] and influence mortality in patients with cancer [5], it is imperative that the complexity of sleep in oncology be recognized and treated in clinical practice [3].

Achieving patient-focused drug development requires clinical trials to include outcomes of importance to patients, and fit-for-purpose assessments to measure those concepts of interest [6]. Therefore, aspects of sleep such as sleep quality, quantity, and impact on health-related quality of life (HRQoL) may be important measurement concepts to monitor and manage in clinical practice, and to assess in clinical trials, to inform understanding of how new treatments affect sleep. Currently, sleep outcomes are rarely explored in clinical trials, likely due to a lack of awareness surrounding the impact of sleep on patient outcomes and the perception that assessment may be difficult or imprecise [7]. However, studies recommend recognition of sleep as a crucial component of cancer care, public health initiatives increasing awareness, and routine screening for sleep disorders in patients with cancer [3]. This could allow early treatment using evidence-based sleep interventions to reduce side effects and improve patient well-being and treatment adherence [3].

Conceptualizing the patient experience of sleep impairment in cancer is a pivotal step to identify clinical outcome assessments, such as patient-reported outcomes which measure sleep impairment and its associated impacts. Ordinarily, this is achieved via the development of a conceptual model; although there is a conceptual model of the patient experience of sleep impairment in breast cancer, it does not provide a comprehensive, holistic overview of sleep impairment across oncology indications or explore how sleep impairment may be linked to measurable functional and HRQoL impacts [8]. Therefore, this study aimed to review the existing literature to comprehensively understand the patient experience of cancer-related sleep impairment.

## 2. Materials and Methods

### 2.1. Eligibility Criteria

Studies with a qualitative methodology that described the patient experience of cancer-related sleep impairment in any oncology indication, published in English between January 2019 and January 2024, were eligible for inclusion. Full details of the eligibility criteria applied to all articles identified within the searches are detailed in Table 1. This was a targeted literature review which aimed to identify up to 25 of the most relevant/informative studies. The targeted approach to literature identification was selected to ensure the most informative studies were included for analysis, based on the review eligibility criteria, coverage across a range of oncology indications, and volume of extractable information to inform development of the conceptual model.

### 2.2. Data Sources, Search Strategy and Quality Assessment

Database search strings were used to identify relevant articles (full publications or conference abstracts) indexed in MEDLINE^®^, Embase, and PsycINFO. Three search strings were developed to account for differences in syntax within each database and combined terms for “cancer” AND “sleep impairment” AND “qualitative research studies” OR “conceptual models”. Supplementary hand-searching of the Food and Drug Administration (FDA) and European Medicines Agency (EMA) websites and the reference lists of any included studies identified from database searches was performed. The quality of included studies was assessed by one author using the Critical Appraisal Skills Programme (CASP) checklist for qualitative studies (Table S1 in the Supplemental Material) [9]. The checklist was designed to be used as an educational pedagogical tool and therefore numerical values were not assigned to the studies, nor were studies excluded based on the critical appraisal results. The most frequently identified issue with publications was inadequate reporting regarding consideration of the relationship between researcher and participant [10,11,12,13,14,15], i.e., if the researcher considered their own role, potential bias, and influence on the study and data collection.

Electronic database searching was conducted on 26th January 2024. All records identified were downloaded into a bespoke Microsoft Access database used to manage citation screening, and duplicates were removed. Studies were screened by their titles and abstracts (first pass) by two authors. Studies that were potentially relevant were obtained in full and reviewed (second pass) by two authors. Discussion with the wider team of authors was used to resolve any discrepancies. A summary of the reasons for exclusion at first and second pass is presented in a Preferred Reporting Items for Systematic Reviews and Meta-Analyses (PRISMA) flow-chart (Figure 1), in accordance with the eligibility criteria presented in Table 1.

### 2.3. Data Extraction and Analysis

Data extraction and analysis of patient quotes or author descriptions/interpretations were conducted by two authors trained in qualitative research techniques. Extracted data underwent secondary analysis using semantic, qualitative, directed content analysis techniques [16,17]. The analysis followed an experiential, realist approach, which focused on participants’ individual perspectives and experiences.

The following five-step process of qualitative directed content analysis was applied [18]:

1. immersion in the data to facilitate researcher understanding of the study and discussion of overarching analytic ideas related to the study objectives,
2. descriptive codes assigned to quotes within the papers and discussed by the project team to support consistent code application, with coded papers quality checked by the project lead at regular intervals for completeness,
3. iterative review of codes throughout the coding stage, with codes merged together or split into more detailed codes as required,
4. defining and refining concepts and domains through research team discussion to reflect on the applied codes and organize coding,
5. reporting of findings.

Coding of extracted data was facilitated by the qualitative analysis software, ATLAS.ti version 9. A conceptual model was developed from the coded data to present a visual illustration of the experience of cancer-related sleep impairment.

**Figure 1:**
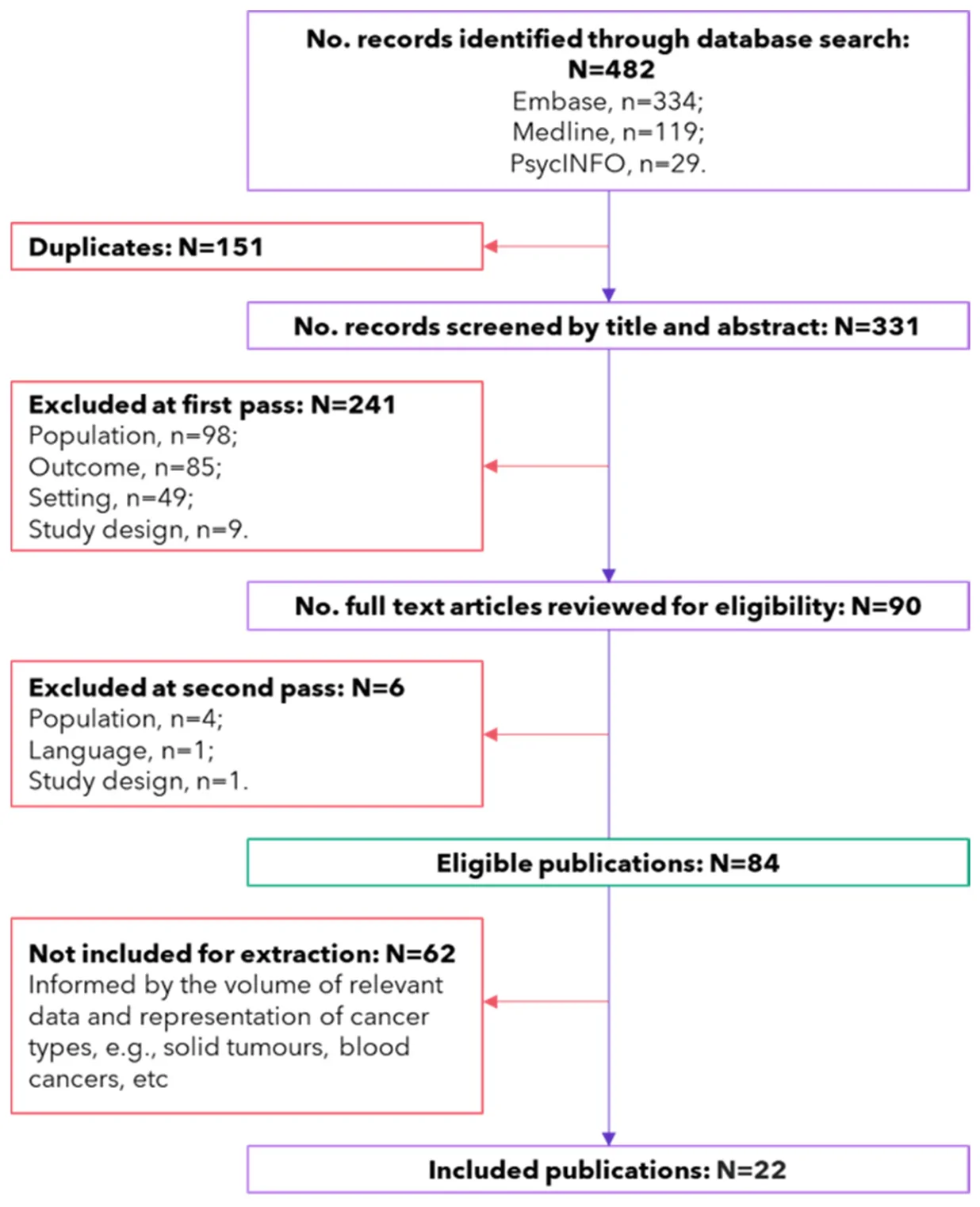
Study selection process.

## 3. Results

### 3.1. Study Selection

The electronic searches yielded 482 records. Following the removal of 151 duplicates, 331 records were screened by title and abstract (*n* = 241 were excluded according to the eligibility criteria). The full-text publications of the remaining records (*n* = 90) were screened (*n* = 6 excluded), and no additional relevant publications were identified via supplementary hand-searching. A total of 84 publications appeared relevant and were eligible for data extraction.

All authors attended a meeting to select the most relevant publications for development of the conceptual model, where publications were reviewed to determine the volume of extractable qualitative data, including patient quotations or author interpretations describing sleep impairment. Publications with more data were prioritized for inclusion, and consideration was given to inclusion of a varied representation of cancer types and stages of the cancer journey. The most frequent reason for study exclusion at this stage was a limited volume of extractable information. Any publications containing a conceptual model of sleep impairment in oncology were retained. Following this meeting, 22 publications were retained for data extraction (Figure 1).

### 3.2. Study Characteristics

The perspectives of 531 patients, 31 caregivers, and 60 healthcare professionals with experience of cancer-related sleep impairment across 22 studies were included in this review. A wide range of oncology indications (*n* = 25 indications) were identified in the included studies, including breast cancer [8,10,15,19,20,21,22,23,24], prostate cancer [20,22,23,24,25,26], and colorectal cancer [20,22,27] (Figure 2). Five studies included a mixed population of multiple cancer indications [15,20,22,23,24]. Studies were most frequently conducted in the United States [8,10,13,14,20,24,27,28,29,30,31] and Europe [13,15,21,22,32], but global representation was obtained, including publications from Canada [23,26], Australia [11,12], China [33], Japan [25], and Taiwan [19]. A summary of the characteristics of the 22 included studies is provided in the Supplementary Material (Table S2).

### 3.3. Sleep Impairment Descriptors

Studies reporting on cancer-related sleep impairment most frequently described a general difficulty sleeping [8,11,12,15,19,20,21,24,26,27,29,30,31,32,33], difficulty falling asleep [8,10,11,13,14,19,20,21,22,24,26,27,28,29], difficulty staying asleep [8,10,11,19,20,24,25,26,27,28,29], poor quality sleep [8,10,11,12,13,19,20,24,26,27,33], and insomnia [10,11,12,19,20,22,23,24,26,28]. Studies also commonly reported an increased need for sleep or difficulty getting enough sleep [8,15,27,29,30], or a disrupted sleep pattern [11,12,19,20,27,31] and reduced sleep duration [8,14,26]. Inconsistent sleep [10,29,30], along with difficulty waking up [21] or sleeping in certain positions [30] was also described (Table S3).

### 3.4. Sleep Impairment Triggers

Triggers for cancer-related sleep impairment included stress [8,10,24], anxiety, worry, or overthinking [8,10,11,13,15,19,20,22,24,25,28,30,33] associated with treatment or diagnosis. Treatment-related experiences, including those associated with chemotherapy (fatigue, tiredness, or anemia) [8,11,15,20,27,31], hormone therapy (vasomotor symptoms or sleep disruption due to night sweats and hot flashes) [8,19,20,21], surgery (sleep positioning or post-operative pain) [8,15,33], steroids (increased alertness/difficulty sleeping) [12], and anticonvulsants (excessive drowsiness) [11,12], also triggered sleep impairment. Cancer-related symptoms, such as discomfort/pain [11,13,15,19,24,26,27,28,33], nocturia [8,11,19,25], and breathing difficulties [15,30], further impacted sleep in some patients. Less commonly reported symptoms that triggered sleep impairment included tingling and cramps [11], stiffness [30], digestive symptoms [30], pruritus [28], restlessness [11], cough [33], headache [11], and daytime hypersomnolence and day-night disruption [12]. External factors also worsened sleep, including environmental disturbances (such as the hospital environment or noise) [11,31], environmental temperature [11]. Poor sleep hygiene (lack of sleep/wake schedule; not having a nightly routine) [10,20], daytime naps [20], tolerance to sleep medications [20], caffeine [20], use of electronic devices [20], bed partner disturbances (such as snoring) [11], and role responsibilities, such as helping others [10] (Table S4).

### 3.5. Impacts

#### 3.5.1. Physiological and Cognitive Function

Patients with cancer-related sleep impairment frequently reported experiencing fatigue, described as increased daytime sleepiness or tiredness [8,10,11,12,22,25,32], exhaustion/ decreased energy [10,21], grogginess [10,24] or fogginess [8]. Amongst these patients, some reported that they would wake up tired, or consistently felt tired regardless of how much sleep they had [8,10,32]. Physical sensations, such as having a headache or pain [11,22] and feeling “unrested” [8], “sick” [22], or “hungover” [20], were also described. The sensation of feeling “hungover” was associated with the use of sleep medications as a coping strategy employed by patients to manage the sleep impairment [20] (Table S5).

Patients also experienced cognitive issues due to cancer-related sleep impairment, including difficulty concentrating [21,22] or following conversations [22], and forgetfulness [22] (Table S6).

#### 3.5.2. Emotional

Patients frequently reported that cancer-related sleep impairment caused worry/stress [11,19,22,28], anxiety [10,20], distress [12,21], and fear [20]. Feelings of anxiety and distress as a result of sleep impairment were described separately from the emotional impact of treatment or diagnosis that could trigger sleep impairment. Patients described anxiety upon waking during the night regarding the potential subsequent impacts on daily functioning if they felt tired the next day [20], as well as experiencing anxiety during the following daytime, due to increased tiredness [10]. Frustration [8,11,21,22], irritation/ snappiness [10,21,22], grumpiness [22], and anger [22] were common and were sometimes generated by the difficulties in day-to-day functioning and the ability to perform role responsibilities caused by sleep impairment, or unsuccessful self-management strategies [22]. Patients also reported personality changes, or not feeling like themselves following the onset of sleep disturbance [22] and feelings of helplessness, depression, and guilt [22] (Table S7).

#### 3.5.3. Activities of Daily Living (ADLs)

Patients frequently reported that cancer-related sleep impairment impacted their general functioning or attending to their needs [8,11,12,21,22,28]. A number of activities were specifically described as being impacted by sleep impairment, including difficulty cleaning [21], getting dressed [21], or going for a walk [11] due to tiredness. Patients reported that activities were also more difficult to complete due to experiencing a lack of motivation [10,22], reduced daytime activity [22], and reduced productivity [10], and that naps were needed to manage the increased tiredness associated with cancer or their treatment experiences [8,11,21,22,26,30] (Table S8).

#### 3.5.4. Social Functioning and Relationships

Patient relationships with others were impacted due to irritability as a result of cancer-related sleep impairment [10,21], and some patients experienced difficulty participating in social events due to feeling tired, or difficulty making social plans as it was harder to commit to attending events due to the unpredictability of sleep [22]. Patients experienced reduced quality time with their partners as they needed to sleep separately to minimize the impact of nighttime awakenings on their partner’s sleep [22], and struggled with the feeling that significant others expected them to be the person they were prior to their cancer diagnosis and did not understand personality changes [22] (Table S9).

#### 3.5.5. Hobbies and Leisure Activities

Cancer-related sleep impairment impacted hobbies and leisure activities, with patients reporting being unable to take part in exercise activities due to a lack of consistent sleep [10] (Table S10).

#### 3.5.6. Work and Financial

Poor sleep led to difficulty completing work tasks [28] and patients needed to reduce their work hours due to sleep impairment affecting their ability to wake up early and concentrate [21], with some patients unable to work at all due to the impact of insomnia [22] (Table S11).

#### 3.5.7. Treatment

Patients considered discontinuing their treatment as their sleep quality was negatively affected by the side effects of hormone therapy, which subsequently impaired their daily functioning [21] (Table S12).

### 3.6. Coping Strategies

Participants reported that they used medication or supplements to manage difficulty sleeping, such as sleep medication, herbal remedies, and medical cannabis [8,10,11,12,19,20,22,23,24,32], or changed the timing of their cancer medication to minimize the impact of side effects on sleep [12,21].

Practicing good sleep hygiene was also reported, including maintaining a consistent bedtime and a nightly wind-down routine [8,10,11,20,22]. Patients avoided or reduced alcohol or caffeine intake [8,20,21,22] and their use of electronic devices, as they were aware of the relationship between screen time and sleep disturbances [8,10,11,20], although some patients reported using caffeine and daytime naps to mitigate the effects of insomnia [22]. Participating in calming or relaxing activities such as breathing exercises [8,11,12,22], or in meditation or prayer [10,11], physical activity during the daytime to encourage sleep at nighttime [10,11], and behavioral or psychological interventions, such as cognitive behavioral therapy for insomnia (CBT-I) [12,23], were reported, as well as maintaining a positive outlook [11] and thinking about plans for the future to distract from the sleep difficulty [11]. Patients managed the environmental temperature [8,10,21] or noise [10] to cope with sleep impairment, and avoided social activities in the evening to reduce mental stimulation [8] (Table S13).

### 3.7. Conceptual Model of the Patient Experience of Sleep Impairment in Oncology

As no existing comprehensive conceptual models depicting the patient experience of sleep impairment in cancer were identified by this study, a conceptual model of the patient experience of cancer-related sleep impairment was developed (Figure 3). The model provides a visual depiction of sleep impairment triggers, descriptors of the way in which sleep impairment is experienced, and the impact of sleep impairment on aspects of patients’ HRQoL. Coping strategies that help prevent triggers and reduce the HRQoL impacts experienced are also represented.

## 4. Discussion

This global review of 22 studies (*n* = 531 patients across 25 indications) conceptualized the patient experience of cancer-related sleep impairment and its associated impacts on HRQoL. The identified concepts, including the descriptors, triggers, and impacts of sleep impairment, were interconnected. Patients reported having difficulty falling or staying asleep, having poor quality sleep or a disrupted sleep pattern, or experiencing insomnia, with similar descriptions of sleep impairment across different indications. The impacts of sleep impairment on patient HRQoL varied and affected multiple domains, including social and relationship functioning, work, hobbies, cognition, emotional wellbeing, treatment experiences, daily activities, and physiological impacts. Patients described how factors such as the emotional impact of diagnosis or treatment, their experience of cancer-related and treatment-related symptoms and side effects, and external factors such as their environment led to impaired sleep. Cancer-related sleep impairment negatively impacted patients’ physiological, cognitive, emotional, and social functioning, as well as their ability to perform ADLs, engage in leisure activities, work, and adhere to treatment regimes. In turn, the impacts on patient HRQoL caused further sleep difficulty, demonstrating the cyclical nature of the overall experience of sleep impairment. It was a “vicious cycle” for patients [11,22], and coping strategies, such as sleep medication or supplements, were utilized to manage both the triggers and impacts of sleep impairment.

This finding is supported by previous studies, which report that poor sleep quality as a result of sleep disruptions and disruptions to circadian rhythms can lead to a variety of psychological and physiological mechanisms, which can in turn lead to the development and persistence of cancer-related fatigue [1]. While empirical testing was beyond the scope of the current work, future research could examine the temporal associations between concepts to further support the causal relationships described in the manuscripts across sleep disturbance triggers, symptoms, and impacts. Sleep disturbances can also affect cancer development and progression due to the impact of sleep on immune function, with studies reporting an association between inadequate sleep or sleep disorders and elevated levels of pro-inflammatory cytokines (e.g., TNF-α and IL-6), which can impair immune surveillance and promote tumor growth [3]. Medication to improve patient sleep, as well as CBT-I and continuous positive airway pressure (CPAP) therapy for obstructive sleep apnea, may reduce inflammation, improve immune responses and, consequently, improve cancer outcomes [3]. In general, sleep-related outcomes are increasingly of interest due to novel sleep measurement approaches. A recent literature review explored the patient perspective of sleep disturbance across a number of therapeutic areas to inform opportunities for meaningful digital measurement of the patient experience of sleep [34]. Based on these findings, a conceptual model of sleep disruption and associated impacts was developed. However, the model does not include data related to patients living with cancer; thus, the work described here addresses an important gap in the sleep literature.

A previous review on cancer-related fatigue by the authors of the current study described a similar, all-encompassing impact across patients’ lives, where associated triggers and impacts were interconnected. Lack of rest/sleep was described as triggering fatigue for patients living with cancer, and patients reported impaired sleep as a key impact of cancer-related fatigue. This review highlighted the need for increased awareness around key symptoms that are important to patients [35]. The results of these studies may help facilitate discussion between patients and clinicians, and aid in selecting the right patient-reported outcomes to effectively measure these symptoms.

Conceptual models represent health experiences that result from conditions such as cancer and can underpin the identification or development of appropriate patient-reported outcome measures to reliably assess concepts such as sleep impairment. Findings from our targeted literature review informed the development of a new conceptual model, which expanded on a recent high-level model developed for sleep impairment in breast cancer [8]. The previous, indication-specific model, which was based on insights from females alone, described a summary experience of sleep during treatment, post-treatment, and throughout the first five years of survivorship [8]. Our newly developed model provides a holistic, concept-level depiction of the patient experience of sleep impairment across sexes, countries, and indications in oncology. Future research should investigate the endorsement of concepts from a patient perspective, through understanding the real-world relevance and applicability of the triggers, symptoms, and impacts for specific patient populations. This may support identification of the core and peripheral components of the patient experience of sleep impairment within a specific oncology context.

The model has important implications for routine clinical practice. For example, it may facilitate improved discussions between patients and clinicians regarding cancer-related sleep impairment experiences [36]. Conceptual models can also represent the potential concepts of interest for assessment in clinical trials, facilitating communication between manufacturers and regulatory reviewers [6]. Therefore, patient-reported outcome decision-making for clinical research should be guided by a conceptual model defining the relevant outcomes of interest and their posited relationships [37]. Whilst the measurement of cancer-related sleep impairment can be challenging, the holistic conceptual model developed in this study can provide a basis for patient-reported outcome selection during early-phase clinical trial planning.

To our knowledge, this is the first conceptual model developed for cancer-related sleep impairment using insights from individuals living with a variety of oncology indications, including both solid and hematological malignancies, at different disease stages across a range of countries. All studies that were reviewed to inform the conceptual model appeared to use appropriate qualitative methodology, according to the quality assessment conducted in this study. When selecting the most relevant publications for development of the conceptual model, selection bias may have been introduced by the selection of a higher volume of publications for certain cancers over others; this limitation was managed by ensuring consideration was given to the varied representation of cancer types. However, the selection of papers also reflects the greater availability of published studies on cancer-related sleep impairment for more prevalent cancer indications. As it was not always possible to identify the indication associated with patient quotes provided in the published literature, and formal sub-group analysis across indications was not conducted, this review did not compare the experience of sleep impairment or its importance to patients across different cancer indications in detail. However, findings indicated that the patient experience of cancer-related sleep impairment and the associated impacts are broadly consistent across oncology indications and treatments. Furthermore, the findings are informed by the geographic and cultural context of original data collection. As only articles with English-language titles and abstracts were eligible for inclusion, there is an inherent risk of publication selection bias, with limited findings regarding the experience of sleep in other settings. In addition, some publications did not clearly report information to understand the quality of study design, data collection, or analysis, which may introduce bias to the original publications that reported data informing the model development. The conceptual model should be applied through a disease-specific and population-specific lens to ensure that the concepts most relevant to the context of use are assessed, and future research could examine any variations across cancer indications by establishing the patient endorsement of specific concepts that may be amenable to treatment interventions. As this study involved a targeted literature review, different methodological approaches, such as a systematic literature review, could also provide additional sources to investigate.

## 5. Conclusions

Cancer-related sleep impairment is an important symptom that frequently affects individuals who are living with cancer and can be triggered by the anxiety associated with cancer, cancer symptoms, treatment side effects, or external contributing factors. Poor sleep has a considerable impact on daily life for patients, which in turn causes further sleep impairment, in a cyclical relationship. There is an unmet need for increased awareness around the importance of sleep in oncology, discussion between patients and clinicians, and treatment for sleep impairment, in order to optimize patient outcomes.

## Figures and Tables

**Figure 2:**
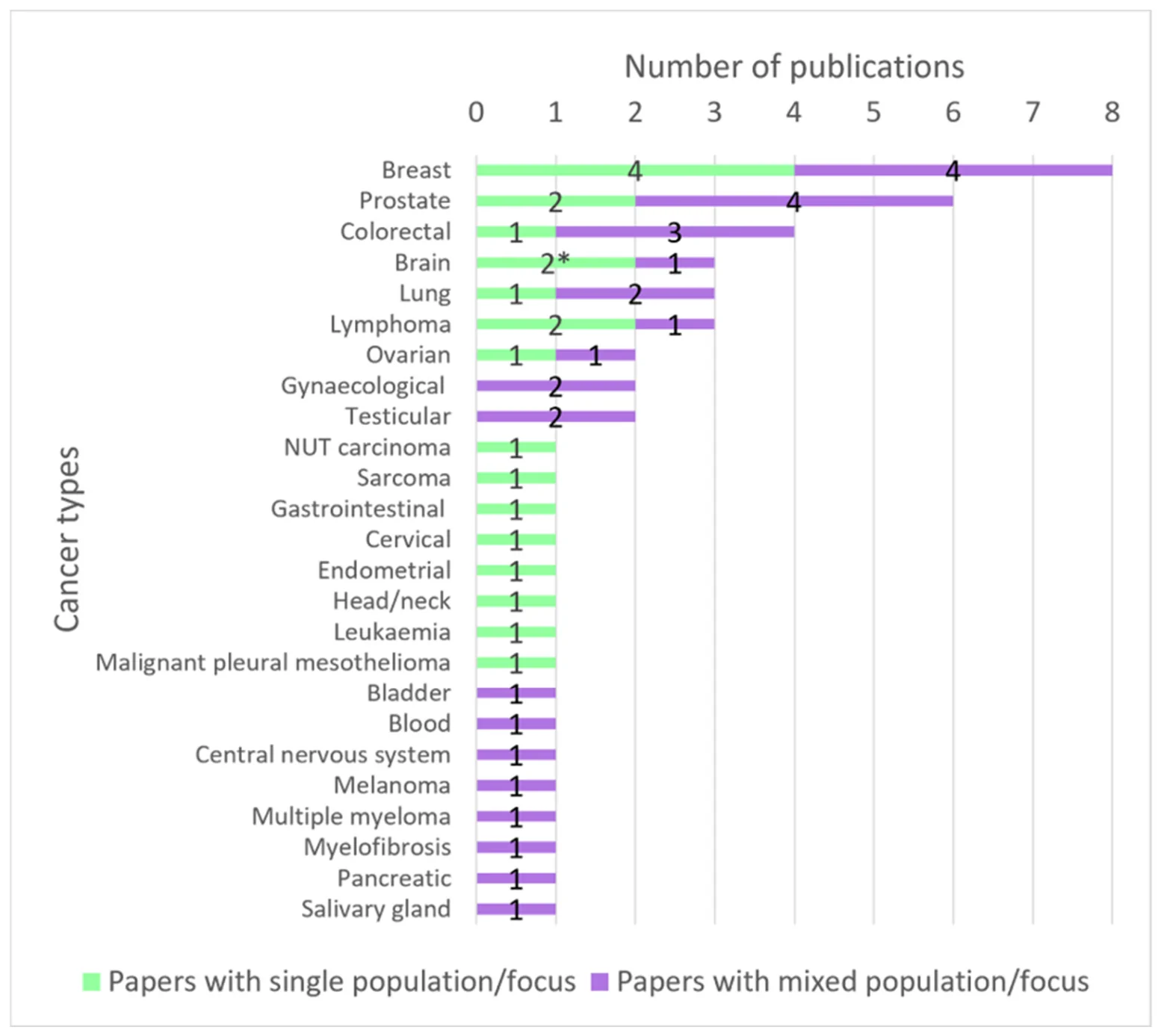
Summary of oncology indications represented by included studies. 1. * n = 21/22 papers enrolled patients; *n* = 1 paper (that focused solely on brain tumors) recruited only HCPs [12]. 2. Abbreviations: NUT, nuclear protein of the testis.

**Figure 3:**
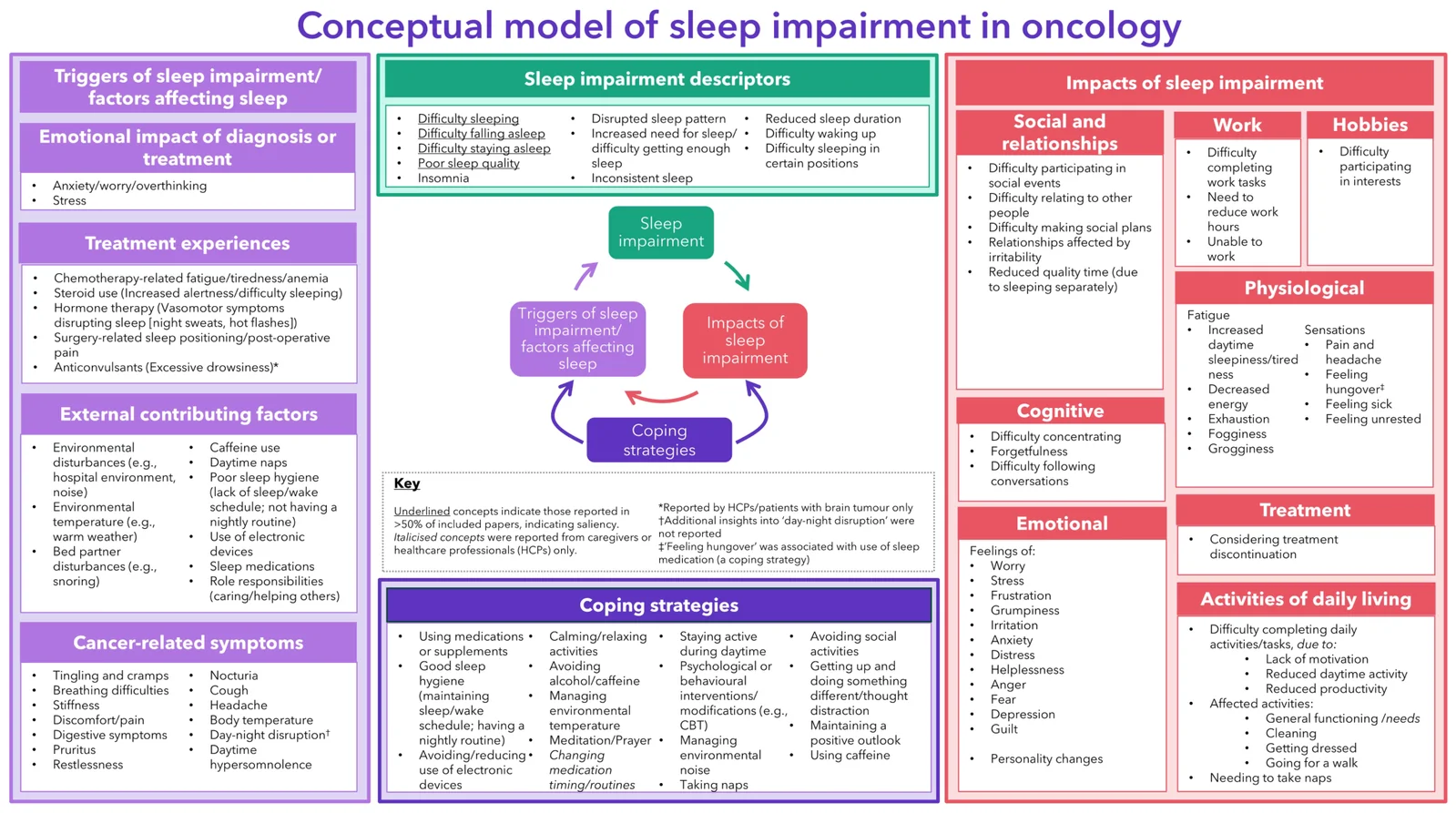
Conceptual model of sleep impairment in oncology.

**Table 1:** Eligibility criteria.

Criteria	Include	Exclude
Population	Adults diagnosed with any type of cancer ^1^ Recent survivors, defined as any of the following: Achieving remission in the last 2 years or less Concluding treatment in the last 2 years or less *Or, if neither of the above were reported in the publication:* Reported to be a survivor/in remission/no longer receiving treatment, AND diagnosed in the last 5 years or less	Studies that enrolled pediatric participants only Studies with mixed populations were eligible if data were reported for adults and pediatric patients separately Long-term survivors (i.e., did not meet definitions provided for ‘recent’ survivors) Studies that enrolled survivors but did not report time since remission/treatment end/diagnosis
Intervention/comparator	No restrictions	No restrictions
Study design	Studies with qualitative methodology (e.g., qualitative interviews or focus groups)	Studies with quantitative methodology only Case reports Review papers were not eligible for inclusion, but highly relevant review papers (i.e., those summarizing qualitative research) were reviewed to determine whether any of the primary studies were eligible for inclusion via handsearching methods
Outcomes	Patient experience of cancer-associated sleep impairment and associated impacts Patient, clinician, or caregiver descriptions of patient’s sleep and associated health-related quality of life impacts Outcomes reported as direct quotes or author summaries of collected data	Patient experience of other symptoms and impacts of cancer or cancer treatment (i.e., not sleep impairment) Quantitative results only (e.g., clinical outcome assessment measure scores) Studies that used multiple-choice survey methodology were flagged for discussion, and highly relevant publications were included if there was a lack of fully qualitative research
Publication language	English Non-English publications with English titles and abstracts	Non-English publications with non-English titles/abstracts
Date of publication	Jan 2019–Jan 2024	Published prior to 2018

^1^ Highly relevant papers that reported findings from qualitative research conducted with clinicians or caregivers were also eligible.

## Data Availability

The datasets generated and/or analyzed during the current study are available from the corresponding author on reasonable request.

## Supplementary Materials

The following supporting information can be downloaded at https://www.mdpi.com/article/10.3390/cancers18193191/s1. Table S1: Qualitative targeted literature review: CASP quality assessment of included studies; Table S2: Summary of studies included (*N* = 22); Table S3: Sleep impairment descriptors identified in the qualitative literature; Table S4: Sleep impairment triggers identified in the qualitative literature; Table S5: Physiological impacts of sleep impairment within the qualitative literature; Table S6: Cognitive impacts of sleep impairment within the qualitative literature; Table S7: Emotional wellbeing impacts of sleep impairment within the qualitative literature; Table S8: Activities of daily living (ADL) impacts of sleep impairment within the qualitative literature; Table S9: Social and relationship impacts of sleep impairment within the qualitative literature; Table S10: Leisure activity impacts of sleep impairment within the qualitative literature; Table S11: Work impacts of sleep impairment within the qualitative literature; Table S12: Treatment impacts of sleep impairment within the qualitative literature; Table S13: Coping strategies used to manage sleep impairment within the qualitative literature.

## Abbreviations

The following abbreviations are used in this manuscript:

CASP	Critical Appraisal Skills Programme
CBT-I	cognitive behavioral therapy for insomnia
CPAP	continuous positive airway pressure
EMA	European Medicines Agency
FDA	Food and Drug Administration
IL-6	Interleukin-6
NUT	Nuclear protein of the testis
PRISMA	Preferred Reporting Items for Systematic Reviews and Meta-Analyses
TNF-α	Tumor Necrosis Factor-alpha
