# Supplementary material for: Sleep Impairment in Oncology: Development of a Novel Patient-Centric Conceptual Model
Source: Cancers (Basel). 2026 Oct 2;18(19):3191. doi: 10.3390/cancers18193191 (PMC13664054; doi:10.3390/cancers18193191)
Supplement: Supplementary file 1 [file cancers-18-03191-s001.zip › cancers-4569065-supplementary.DOC.pdf]

Supplementary Information: Sleep impairment in oncology; development of a novel patient-centric conceptual model

Harriet Makin [1], Sophie Van Tomme [2], Natalie VJ Aldhouse [1], Samantha Wratten [1], Helen Kitchen [1], Chloe Carmichael [1], Cecile Gousset [3], Paul Cordero [4]

1) Clinical Outcomes Assessment, Clarivate Analytics, 70. St Mary Axe, London, United Kingdom, EC3A 8BE
2) Patient-Informed Development & Health Value Translation, Research & Development, Sanofi, Paasheuvelweg 25, 1105BP, Amsterdam, The Netherlands
3) Patient-Informed Development & Health Value Translation, Research & Development, Sanofi, 82 Avenue Raspail, 94255, Gentilly, France
4) Patient-Informed Development & Health Value Translation, Research & Development, Sanofi, 410 Thames Valley Park Dr, Earley, Reading, United Kingdom, RG6 1PT

Contents of file: Supplementary tables reporting results of quality assessment, overview of sleep impairment descriptors, triggers, and impacts to health-related quality of life, as reported in the literature through direct patient reports and author summaries.

1.1. Quality Assessment

Table S1 provides an overview of the CASP quality appraisal conducted for the included studies. As part of the CASP appraisal, the reviewer is asked to record a "yes" (✓), "no" (x) or "can't tell" (?) to each prompt. The checklist was designed to be used as a pedagogic tool and therefore the authors do not recommend formally scoring studies/publications.

Table S1. Qualitative targeted literature review: CASP quality assessment of included studies

**Publication**	Was there a clear statement of the aims of the research?	Is a qualitative methodology appropriate?	Was the research design appropriate to address the aims of the research?	Was the recruitment strategy appropriate to the aims of the research?	Was the data collected in a way that addressed the research issue?	Has the relationship between researcher and participants been adequately considered?	Have ethical issues been taken into consideration?	Was the data analysis sufficiently rigorous?	Is there a clear statement of findings?	How valuable is the research?
Bhat, 2021[1]	✓	✓	✓	✓	✓	✓	✓	✓	✓	✓
Chang, 2023[2]	✓	✓	✓	✓	✓	✓	✓	✓	✓	✓
Cheng, 2022[3]	✓	✓	✓	✓	✓	✓	✓	✓	✓	✓
Garland, 2019[4]	✓	✓	✓	✓	✓	✓	?	✓	✓	✓
Geiss, 2022[5]	✓	✓	✓	✓	✓	?	✓	✓	✓	✓
Guillemin, 2022[6]	✓	✓	✓	✓	✓	✓	✓	✓	✓	✓
Hwang, 2023[7]	✓	✓	✓	✓	✓	✓	✓	✓	✓	✓
Ibrar, 2022[8]	✓	✓	✓	✓	✓	✓	✓	✓	✓	✓
Jeon, 2021a[9]	✓	✓	✓	✓	✓	?	✓	✓	✓	✓

Publication	Was there a clear statement of the aims of the research?	Is a qualitative methodology appropriate?	Was the research design appropriate to address the aims of the research?	Was the recruitment strategy appropriate to the aims of the research?	Was the data collected in a way that addressed the research issue?	Has the relationship between researcher and participants been adequately considered?	Have ethical issues been taken into consideration?	Was the data analysis sufficiently rigorous?	Is there a clear statement of findings?	How valuable is the research?
Jeon, 2021b[10]	✓	✓	✓	✓	✓	?	✓	✓	✓	✓
Martin, 2020[11]	✓	✓	✓	✓	✓	?	✓	✓	✓	✓
Nathwani, 2022[12]	✓	✓	✓	✓	✓	?	✓	✓	✓	✓
Nishimura, 2021[13]	✓	✓	✓	✓	✓	✓	✓	✓	✓	✓
Parthipan, 2023[14]	✓	✓	✓	✓	✓	✓	✓	✓	✓	✓
Pompilus, 2021[15]	✓	✓	✓	✓	✓	✓	✓	✓	✓	✓
Reynolds-Cowie, 2020[16]	✓	✓	✓	✓	✓	✓	?	✓	✓	✓
Savard, 2022[17]	✓	✓	✓	✓	✓	✓	✓	✓	✓	✓
Van de Wal, 2023[18]	✓	✓	✓	✓	✓	✓	✓	✓	✓	✓
Van Eck, 2021[19]	✓	✓	✓	✓	✓	?	✓	✓	✓	✓
Walker, 2022[20]	✓	✓	✓	✓	✓	✓	✓	✓	✓	✓

Publication	Was there a clear statement of the aims of the research?	Is a qualitative methodology appropriate?	Was the research design appropriate to address the aims of the research?	Was the recruitment strategy appropriate to the aims of the research?	Was the data collected in a way that addressed the research issue?	Has the relationship between researcher and participants been adequately considered?	Have ethical issues been taken into consideration?	Was the data analysis sufficiently rigorous?	Is there a clear statement of findings?	How valuable is the research?
Yang, 2023[21]	✓	✓	✓	?	✓	✓	✓	✓	✓	✓
Zhou, 2022[22]	✓	✓	✓	✓	✓	✓	✓	✓	✓	✓

1.2. Study Selection

A summary of the characteristics of the studies included in this review is provided in Table S2.

Table S2. Summary of studies included (N=22)

Study	Sample size, N	Cancer type(s)	Males, n	Age (mean [range][a], years)	Country
Bhat, 2021[1]	18 patients	Cutaneous T-cell lymphoma	8	Median 61 [52–70]	USA
Chang, 2023[2]	26 patients	Breast cancer	0	46	Taiwan
Cheng, 2022[3]	18 patients	Diffuse large B-cell lymphoma	11	60 [33–80]	USA
Garland, 2019[4]	63 patients	Mixed population (breast cancer, n=22; prostate cancer, n=13; colon/rectal cancer, n=4; head/neck cancer, n=2; melanoma, n=4;	26	61	USA

Study	Sample size, N	Cancer type(s)	Males, n	Age (mean [range][a], years)	Country
		lymphoma, n=7; leukemia, n=3; lung cancer, n=4; other cancer [not reported], n=13)			
Geiss, 2022[5]	10 patients	Breast cancer	0	54	USA
Guillemin, 2022[6]	25 patients	Metastatic colorectal cancer	8	52 [26–72]	USA
Hwang, 2023[7]	35 patients	Breast cancer	0	44	USA
Ibrar, 2022[8]	25 patients	Breast cancer	0	48 [31–67]	Scotland
Jeon, 2021a[9]	24 patients, 14 caregivers	Malignant brain tumors (glioma)	14	53	Australia
Jeon, 2021b[10]	11 HCPs	Malignant brain tumors (glioma)	3	Not reported	Australia
Martin, 2020[11]	64 patients	Ovarian cancer	0	Europe: 54<br>USA: 62	USA, UK, France, Germany
Nathwani, 2022[12]	22 patients	Relapsed and/or refractory multiple myeloma	13	69	USA
Nishimura, 2021[13]	20 patients	Non-metastatic castration-resistant prostate cancer	20	Median 77 [62–92]	Japan
Parthipan, 2023[14]	36 patients	Metastatic prostate cancer	36	Chemotherapy group: 73<br>ARAT group: 76<br>Radium-223 group: 80	Canada

Study	Sample size, N	Cancer type(s)	Males, n	Age (mean [range][a], years)	Country
Pompilus, 2021[15]	10 patients 17 caregivers	Nuclear protein of the testis carcinoma	5	39	USA
Reynolds-Cowie, 2020[16]	27 patients	Mixed population (breast cancer, n=12; prostate cancer, n=7; colorectal cancer, n=7; gynecological cancer, n=1)	11	62	UK
Savard, 2022[17]	10 patients, 43 HCPs	Mixed population (breast cancer, n=6; prostate cancer, n=1; gynecological cancer, n=1; central nervous system cancer, n=1; salivary gland cancer, n=1)	1	60 [47–74]	Canada
Van de Wal, 2023[18]	24 patients, 6 HCPs	Gastrointestinal stromal tumors	14	59.1 [38–82]	UK
Van Eck, 2021[19]	23 patients	Mixed population (thoracic sarcoma, n=19; breast sarcoma, n=4)	8	Thoracic sarcoma: 5<br>Breast sarcoma: 60	UK, Germany, Norway, The Netherlands, Italy, Cyprus
Walker, 2022[20]	7 patients	Malignant pleural mesothelioma	4	72 [58–79]	USA
Yang, 2023[21]	20 patients	Lung cancer	9	51	China

Study	Sample size, N	Cancer type(s)	Males, n	Age (mean [range][a], years)	Country
Zhou, 2022[22]	24 patients	Mixed population (including breast cancer, prostate cancer, bladder cancer, endometrial cancer, testicular cancer, brain cancer, cervical cancer, blood cancer)<br>*Indication sample sizes not reported.*	8	Median 57 [30–71]	USA

a Extracted where available

Abbreviations: HCP, healthcare professional.

1.3. Sleep Impairment Descriptors

A summary of the types of sleep impairment described in the literature is presented in Table S3.

Table S3. Sleep impairment descriptors identified in the qualitative literature

Sleep impairment descriptor	Number of sources	Sources	Supporting quote(s)
Difficulty sleeping	15	[2-4, 6-10, 14, 15, 18-22]	– *"I just always have trouble sleeping."[3]* [Lymphoma]<br>– Author commentary: Several participants acknowledged that they had difficulty sleeping prior to their cancer diagnosis but that impact of stress related to the cancer diagnosis or the effects of cancer treatments made a pre-existing problem worse.[4] [Mixed populations]<br>– *"I don't know what happens, but it's a bit like your brain has forgotten how to sleep."[8]* [Breast cancer]
Difficulty falling asleep	14	[1-9, 11, 12, 14, 16, 22]	– "[Cutaneous T-cell lymphoma] *doesn't let you sleep at night because you're just constantly fixated on what's going wrong, what could go wrong, how it affects the people that are close to you, all those kinds of things."[1]* [Lymphoma]

Sleep impairment descriptor	Number of sources	Sources	Supporting quote(s)
			– *"I just have to have something like that to make me fall asleep… Some background noise."[5]* [Breast cancer]<br>– *"Falling asleep was hard and terrible."[7]* [Breast cancer]<br>– *"[…] I think I can't fall asleep because then I'm becoming so anxious about not being able to wake up the next morning and being tired the next day."[4]* [Breast cancer]
Difficulty staying asleep	11	[1-7, 9, 13, 14, 22]	– *"Sometimes I wake up halfway through my sleep at night because I always have so many thoughts that I can't let go of."[2]* [Breast cancer]<br>– *"I do wake up in the middle of the night about 3 or 4 times a month just wide awake."[3]* [Lymphoma]<br>– Author commentary: The overarching theme, "No longer able to sleep through the night", was characterized by trouble falling asleep, waking up during the night, and not feeling rested.[7] [Breast cancer]<br>– *"I wake up in the middle of night sometimes with the pain... It feels like needles sticking in your body... And sometimes it's so bad, like I said, I wake up with it."[1]* [Lymphoma]
Poor sleep quality	11	[2, 4-7, 9-11, 14, 21, 22]	– Author commentary: Barriers to high quality sleep. Participants described their sleep as "inconsistent," "interrupted," and "not entirely restful."[5] [Breast cancer]<br>– Author commentary: Participants reported poor sleep quality, noted as difficulty getting up in the morning, and feeling tired upon waking.[9] [Brain tumor]<br>– *"Patterns of sleep disturbance change as disease advances, there's quite a lot of sleep disturbance towards the end. But it's different to the experience at the beginning, it's not that people aren't sleeping, their quality of sleep is really poor, and they're often sleeping at odd times, which makes it hard to maintain."[10]* [Brain tumor]<br>– *"I would cough for a long time. It also affected my quality of sleep at night." [21]* [Lung cancer]

**Sleep impairment descriptor**	**Number of sources**	**Sources**	**Supporting quote(s)**
Insomnia	10	[1, 2, 4, 5, 9, 10, 14, 16, 17, 22]	– Author commentary: For those that reported cancer was the trigger for insomnia, the difficulty sleeping was mainly triggered by the emotional impact of the diagnosis, the impact of hospitalization, or treatments such as chemotherapy or radiation. *"I would probably say right around the time – well, after the diagnosis. But, you know, your mind is racing at that point in time thinking about what could be and always thinking about anticipating the worst and so forth. […]. But then, after going through the chemo and radiation and still […] this inability to go to sleep […]."[4]* [Breast cancer]<br>– Author commentary: The impact of insomnia was significant for all participants. Indeed, many (n=18) reported that the consequences of poor sleep were often more overwhelming than the impact of cancer treatment.[16] [Mixed/indication not specified]<br>– Author commentary: Symptoms related to sleep disturbance were described as insomnia […].[14] [Prostate cancer]
Disrupted sleep pattern	6	[2, 4, 6, 9, 10, 20]	– *"[…] we're almost a year out and there's still this inability to go to sleep or resort back to what the sleeping or behaviour patterns were prior to. So that's when I took notice of my sleep patterns and I did talk to my primary care physician."[4]* [Breast cancer]<br>– Author commentary: Fatigue had implications for patients' sleep patterns […].[6] [Colorectal cancer]<br>– Author commentary: However, some patients described their sleep-wake time had shifted earlier than pre-diagnosis.[9] [Brain tumor]
Increased need for sleep/ difficulty getting enough sleep	5	[3, 6, 7, 15, 19]	– [During/immediately after CAR-T] *"I required a lot of extra sleep, at least 10 or 11 hours, and I normally never slept more than 7."[3]* [Lymphoma]<br>– *"I am not satisfied. It's (sleep) just not enough."[7]* [Breast cancer]<br>– *"He would sleep and sleep and sleep, and I would have to wake him up."[15]* [Caregiver, NUT carcinoma]
Inconsistent sleep	3	[3, 5, 15]	– Author commentary: Participants described their sleep as "*inconsistent*", "*interrupted*", and "*not entirely restful*."[5] [Breast cancer]

**Sleep impairment descriptor**	**Number of sources**	**Sources**	**Supporting quote(s)**
Reduced sleep duration	3	[7, 12, 14]	– *"Your energy level is up, you don't sleep that night much, you're wired, you can't sleep, your brain just keeps going."[12]* [Multiple myeloma]<br>– Author commentary: Most reported sleeping less after treatment.[14] [Prostate cancer]
Difficulty waking up	1	[8]	– Author commentary: […] they struggled to wake up early […].[8] [Breast cancer]
Difficulty sleeping in certain positions	1	[15]	– *"[…] the way I sleep at night are all impacted by that stiffness in my neck."[15]* [NUT carcinoma]

Abbreviations: CAR-T, chimeric antigen receptor t-cell therapy; NUT, nuclear protein of the testis.

1.4. Sleep Impairment Triggers

Table S4 provides an overview of the sleep impairment triggers reported in the literature.

Table S4. Sleep impairment triggers identified in the qualitative literature

Trigger of sleep impairment	Number of sources	Sources	Supporting quote(s)
**Emotional impact of diagnosis or treatment**			
Anxiety/worry/ overthinking	13	[1, 2, 4, 5, 7, 9, 11, 13, 15, 16, 19, 21, 22]	– *"One of the things I noted is I can't shut down my thoughts. My thoughts just race through the night. So, I do a lot of thinking and processing of issues and replaying events, and my mind is too alert. It just reprocesses everything that's happened, either that day, two weeks ago, the day before. What I could've done, should've done."[4]* [Ovarian cancer]<br>– *"The fact that I am dying, I found it a bit confronting at times, you know. So, there's always a level of anxiety and worry and which is better on some days and worse on others."[9]* [Brain tumor]<br>– *"It was difficult for me to sleep through the night…anxiety."[15]* [NUT Carcinoma]<br>– *"It's the stress of it… it's the fact that you're worrying about it, it's how am I going to get up tomorrow morning, and how am I going to get out, and how am I going to get through work."[16]* [Indication not reported]
Stress	3	[5, 7, 22]	– Author commentary: Everyday life stressors and physical and psychological side effects associated with cancer treatment influenced their sleep experience.[7] [Breast cancer]
**Treatment experiences**			
Chemotherapy-related fatigue/tiredness/ anemia	6	[4, 6, 7, 9, 19, 20]	– Author commentary: Physical side effects of cancer treatment were major contributors of sleep disturbance. There was a temporal relationship with a high level of fatigue during treatment. Some slept a lot because they were tired while others were not able to sleep even though they were tired.[7] [Breast cancer]<br>– *"I'm sleeping alright, especially when I stopped the chemo. Now for two or three weeks I'm off these things, I'm fine."[9]* [Brain tumor]

Trigger of sleep impairment	Number of sources	Sources	Supporting quote(s)
Hormone therapy (Vasomotor symptoms disrupting sleep [night sweats, hot flashes])	4	[2, 4, 7, 8]	– Author commentary: Following primary treatment for breast cancer, the onset of adjuvant endocrine therapy resulted in vasomotor symptoms, specifically hot flashes and night sweats, to which participants attributed their sleep disturbance.[4] [Breast cancer]<br>– *"After 10 days of hardly sleeping* [due to hormone therapy]*, I was banging my head on the wall […]."[8]* [Breast cancer]<br>– Author commentary: The most frequent and severe side effects reported across both types of HT (hormone therapy) were sleep disturbance.[8] [Breast cancer]
Surgery-related sleep positioning/ post-operative pain	3	[7, 19, 21]	– Author commentary: Several participants attributed their sleep disturbance to the discomfort due to the side effects of surgery and chemotherapy. Common reasons were challenges in position in bed due to shoulder issues or incision sites from surgical reconstruction, the complication of lymphedema, and pain related to chemotherapy-induced peripheral neuropathy. Trying to find a comfortable position resulted in fragmented sleep.[7] [Breast cancer]<br>– Author commentary: […] one breast sarcoma patient mentioned having trouble sleeping due to limited positions to sleep in after surgery […].[19] [Breast sarcoma]<br>– *""Given the right side trauma, which is the side I used to normally sleep on, […] I started having a lot of trouble (sleeping)."* [Breast sarcoma]
Anticonvulsants (excessive drowsiness) [a]	2	[9, 10]	– Author commentary: When patients were using corticosteroids or anticonvulsants implicated in fatigue, this triggered HCPs to enquire about sleep disturbance specifically. [10] [Brain tumor]
Steroid use (Increased alertness/difficulty sleeping)	1	[10]	– Author commentary: Early in the diagnosis and treatment trajectory, insomnia and heightened alertness at night induced by dexamethasone were the most common types of sleep disturbance noted by HCPs.[10] [Brain tumor]
**External contributing factors**			

Trigger of sleep impairment	Number of sources	Sources	Supporting quote(s)
Environmental disturbances (e.g., hospital environment, noise)	2	[9, 20]	– *"Being in the hospital is torture. You can't get a good night's sleep."[20]* [Pleural mesothelioma]
Poor sleep hygiene (lack of sleep/wake schedule; not having a nightly routine)	2	[4, 5]	– Author commentary: Importantly, participants identified a number of perpetuating behavioural factors that are common targets in CBT-I. These included […] poor sleep hygiene. [4] [Mixed population]<br>– *"I think the main thing… would be to identify the cause of not having a good sleep hygiene program. That would be number one…I like the fact that they give you a lot of information about potential problems you may be having, and it gives you the opportunity to evaluate yourself and see where you fit in."[5]* [Breast cancer]
Environmental temperature (e.g., warm weather)	1	[9]	– Author commentary: Participants described a range of factors as contributors to sleep disturbance, from external and seasonal disturbances (warm weather, noise, snoring, and other disturbance from bed partner) […].[9] [Brain tumor]
Bed partner disturbances (e.g., snoring)	1	[9]	
Caffeine use	1	[4]	– *"Many participants reported an awareness of engaging in behaviors conducive with good sleep hygiene such as not having caffeine too late […], but several others did not realize the potential for these factors to impact their sleep. "But most of the time when it was time to go to bed I'm usually out on my computer. And I put on my computer, I have a cup of tea or something like that. So it's just a habit I guess. And so that hasn't really gone away. I still have a cup of tea late at night. And tea – I don't think the caffeine or whatever is in there prohibits me from getting sleep. It's kind of soothing."[4]* [Prostate cancer]
Daytime naps	1	[4]	– *"And then the other thing is I take a nap every day. I think I told you. Most days I do. And sometimes I feel as though when I don't take a nap I sleep better at night sometimes."[4]* [Prostate cancer]

Trigger of sleep impairment	Number of sources	Sources	Supporting quote(s)
Use of electronic devices	1	[4]	– *"Usually I sort of play something on my phone to sort of make myself sleepy since I get in bed and then you're wide awake. It's the opposite of my husband who hits the bed and is snoring. So sometimes when I do that, I get engaged in what I'm doing on the phone and then I end up staying up even later because – I don't know, then I'm not tired and all of a sudden – eventually get tired and fall asleep. And that's certainly not good because I get up about quarter to five, 4:30 – 5:00, every day."[4]* [Breast cancer]
Tolerance to sleep medications	1	[4]	– *"The Ativan usually – I'll sleep pretty well when I take that as long as I don't take it multiple nights. I find that if I take it one or two, maybe three nights in a row, that usually is pretty good. But if I've been taking it more than that, I find that the impact is lessened. It's like probably a tolerance or something."[4]* [Pancreatic cancer]
Role responsibilities (caring/helping others)	1	[5]	– *"Sometimes when there's a crisis or some things that are going on, it needs to be taken care of… a lot of people do look up to me… That can be something on my mind before I go to sleep."[5]* [Breast cancer]
**Cancer-related symptoms**			
Discomfort/pain	9	[1, 2, 6, 9, 11, 14, 19, 21, 22]	– *"I have had cancer on my chest wall which is like right around my ribcage on my right side, so… I couldn't get comfortable because of the cancer there."[6]* [Colorectal cancer]<br>– *The pain is so intense, especially in my joint, knee joints particularly, and during the treatment, the first four, five days of the treatment, the pain in my knee joints, my ankles, and my feet was so intense I could not sleep at all."[14]* [Prostate cancer]<br>– Author commentary: Patients reported difficulty falling and staying asleep due to […] pain […].[1] [Lymphoma]
Nocturia	4	[2, 7, 9, 13]	– *"Other than waking up for the specific reason of needing to go to the bathroom, I usually have to use the bathroom at least once a night, sometimes twice."[7]* [Breast cancer]

Trigger of sleep impairment	Number of sources	Sources	Supporting quote(s)
			– Author commentary: Frequent nocturnal urination was given as a reason for waking at night […].[9] [Brain tumor]<br>– *"[Getting up] twice is bearable. But when it's up to 3 to 4 times during the night, it's more difficult to get back to sleep, I'm awake longer and I get sleepy during the day."[13]* [Prostate cancer]<br>– *"During the night, after I woke up to go to the toilet and got back into bed and tried to fall back asleep, often, I would almost immediately have to go to the toilet again."[13]* [Prostate cancer]
Breathing difficulties	2	[15, 19]	– *"It is as if I am not getting enough oxygen. I'm a little bit scared about that or panicky sometimes. It is annoying. Normally during the day, I have no problems with it, nor do I suffer from it. But when I am in bed, I am paying attention to it in a different way'."[19]* [Thoracic sarcoma]
Tingling and cramps	1	[9]	– *"[…] have had tingling feet, extremities really, that has a time if you wake at night, getting quite strong cramps in my leg and hands."[9]* [Brain tumor]
Stiffness	1	[15]	– Author commentary: Sleep: Difficulty sleeping related to: […] Symptoms [e.g. […] stiffness […].[15]
Digestive symptoms	1	[15]	– Author commentary: Sleep: Difficulty sleeping related to: […] Symptoms [e.g. […] digestive symptoms […].[15]
Pruritus	1	[1]	– *"Middle of the night, I wake up itching, so it's like every day, all day… Last night, I woke up, like, five times itching." [1]* [Lymphoma]
Restlessness	1	[9]	– Author commentary: Some patients described night-time restlessness, both psychological and physical, as a reason for getting out of bed and moving around at home.[9] [Brain tumor]
Cough	1	[21]	– *"After the operation, I couldn't take a deep breath. Deep breathing would stimulate my cough, and I would cough for a long time. It also affected my quality of sleep at night. I hope that my cough will*

Trigger of sleep impairment	Number of sources	Sources	Supporting quote(s)
			be relieved and I will be able to sleep more soundly."[21] [Lung cancer]
Headache	1	[9]	– Author commentary: Participants described a range of factors as contributors to sleep disturbance […] symptoms of BT ([…] pain […]).[9] [Brain tumor]
Day-night disruption [b]	1	[10]	– Author commentary: However, day-night disruption and daytime hypersomnolence were observed in advanced BT.[10] [Brain tumor]
Daytime hypersomnolence			

a Reported by patients with brain tumor or HCPs only. b Additional insights into 'day-night disruption' were not reported.

1.5. Impacts on HRQoL

1.5.1. Physiological and Cognitive Function

Table S5 provides an overview of the physiological impacts associated with sleep impairment.

Table S5. Physiological impacts of sleep impairment within the qualitative literature

Impact	Number of sources	Sources	Supporting quote(s)
**Fatigue**			
Increased daytime sleepiness/ tiredness	7	[5, 7, 9, 10, 13, 16, 18]	– *"I already had sleep issues before I had cancer. But after… it's worse. I'm always tired."[5]* [Breast cancer]<br>– *"I can get the full eight, even up to nine hours 'cause I've tried. Even then, I'm still tired."[7]* [Breast cancer]<br>– *"Fatigue from chemo can also affect, increasing daytime sleepiness and decreasing night-time sleep."[10]* [Brain tumour]<br>– *I get a good night's sleep I'll wake up tired."[18]* [Gastrointestinal stromal tumour]
Exhaustion/ decreased energy	2	[5, 8]	– Author commentary: Sleeplessness impacted participants' ability to cope well during the day and they became irritable, often snapping at family and friends due to exhaustion.[8] [Breast cancer]<br>– Author commentary: Participants commonly described how sleep disturbance resulted in […] feeling […] *"exhausted"* during the day. [Breast cancer]
Grogginess	2	[5, 22]	– Author commentary: […] this can result in daytime dysfunction (i.e., morning grogginess).[5] [Breast cancer]<br>– *"With the [zolpidem], I still, sometimes, would wake up and when I'd wake up in the morning I would be very groggy…"[22]* [Breast cancer]
Fogginess	1	[7]	– Author commentary: Some participants reported feeling tired, foggy, and not rested in the morning, even those who perceived having a sufficient sleep.[7] [Breast cancer]
**Sensations**			

**Impact**	**Number of sources**	**Sources**	**Supporting quote(s)**
Pain and headache	2	[9, 16]	– *"Ah, a lot of times I'd love to sleep in little bit later, but I can't, my body just doesn't want to sleep in, I just wake up because I've headache and I want to stay in bed, and sometimes I just do have to because I'm not feeling 100%, but generally, nah... I wake up tired."[9]* [Brain tumor]<br>– Author commentary: Sleep disruption had a considerable impact on physical well-being. Symptoms such as a '*heightened pain level within your body*', '*thumping headaches*', and feeling '*physically sick*' were reported by the majority of participants. They also highlighted how lack of sleep tended to '*exaggerate*' the experience of these difficulties.*[16]* [Mixed population]
Feeling hungover [a]	1	[4]	– *"And even if I take an Ativan, which is what my oncologist encouraged me to do if I can't fall asleep, I have – I feel hungover – or some sort of feeling the next day which makes it not worth it to me."[4]* [Breast cancer]
Feeling sick	1	[16]	– *Author commentary: Sleep disruption had a considerable impact on physical well-being. Symptoms such as a 'heightened pain level within your body', 'thumping headaches', and feeling 'physically sick' were reported by the majority of participants. They also highlighted how lack of sleep tended to 'exaggerate' the experience of these difficulties.[16] [Mixed population]*
Feeling unrested	1	[7]	– Author commentary: The overarching theme, "No longer able to sleep through the night," was characterized by trouble falling asleep, waking up during the night, and not feeling rested.[7] [Breast cancer]

[a]'Feeling hungover' was associated with use of sleep medication (a coping strategy).

Table S6 presents an overview of the impacts of sleep impairment on cognitive function.

Table S6. Cognitive impacts of sleep impairment within the qualitative literature

Impact	Number of sources	Sources	Supporting quote(s)
Difficulty concentrating	2	[8, 16]	– Author commentary: Cognitive impairments were also frequently highlighted as a problematic consequence of poor sleep. Forgetfulness, difficulty concentrating, and an impaired ability to follow conversations in social and occupational settings were most common. These impairments only served to exacerbate participants' difficulties in social and interpersonal functioning, in addition to precluding the enjoyment of previously pleasurable activities.[16] [Mixed population]<br>– *"I can't concentrate, I can't hold a thought, I can't remember what that person said to me three minutes ago...yeah, memory, concentration, conversations, sociability, everything's affected'."[16]* [Mixed population, indication not specified]
Forgetfulness	1	[16]	
Difficulty following conversations	1	[16]	

1.5.2. Emotional

Table S7 provides an overview of how sleep impairment impacted emotional well-being, as reported in the literature.

Table S7. Emotional wellbeing impacts of sleep impairment within the qualitative literature

Impact	Number of sources	Sources	Supporting quote(s)
**Feelings of:**			
Worry/stress	4	[1, 2, 9, 16]	– *"Insomnia, secondary to other clinical symptoms and worry about disease* [Cutaneous T-cell lymphoma] *doesn't let you sleep at night because you're just constantly fixated on what's going wrong, what could go wrong, how it affects the people that are close to you, all those kinds of things."[1]* [Lymphoma]<br>– *"I guess I probably out of a week I would have a couple of nights of fairly sound sleep, which gets me through the week. […] It's frustrating as much as anything. It's still stressful, it's still always there, but you learn to deal with it."[9]* [Brain tumor]

**Impact**	**Number of sources**	**Sources**	**Supporting quote(s)**
Frustration	4	[7-9, 16]	– Author commentary: Women were frustrated by not being able to get enough hours of sleep.*[7]* [Breast cancer]<br>– Author commentary: There was a patent and powerful sense of frustration at the persistence of sleep difficulties, which was intensified by a feeling of pressure to return to 'pre-cancer' family and occupational roles from others, who could not understand or empathize with the pervasive impact of chronically disturbed sleep.[16] [Mixed population]<br>– *"I guess I probably out of a week I would have a couple of nights of fairly sound sleep, which gets me through the week. […] It's frustrating as much as anything."[9]* [Brain tumor]
Irritation/ snappiness	3	[5, 8, 16]	– Author commentary: Sleeplessness impacted participants' ability to cope well during the day and they became irritable, often snapping at friends and family due to exhaustion.[8] [Breast cancer]<br>– Author commentary: In addition, some participants reported that a lack of restful sleep affected their daytime mood, including increased levels of daytime anxiety and irritability toward others. This, in turn, affected social functioning and relationships.[5] [Breast cancer]
Anxiety	2	[4, 5]	– Author commentary: Participants reported that the inability to sleep came with its own form of anxiety, compounded by watching the clock in the middle of the night and worrying about the impact of poor sleep on their ability to function the next day.*[4]* [Mixed population]<br>– *"My anxiety's kicking because I'd be tired, and every little thing agitates me… I tend to get anxious a lot and I get on people's nerves because I'm tired."[5]* [Breast cancer]
Distress	2	[8, 10]	– *"After 10 days of hardly sleeping, I was banging my head on the wall on the landing, screaming, even during the day."[8]* [Breast cancer]<br>– Author commentary: The recognised abnormal day-night patterns cause distress in both patients and caregivers.*[10]* [Brain tumor]

Impact	Number of sources	Sources	Supporting quote(s)
Grumpiness	1	[16]	– Author commentary: Indeed, these changes impacted on close personal relationships with people becoming 'irritable', 'snappy', 'grumpier' […]."[16] [Mixed population]
Helplessness	1	[16]	– Author commentary: Although some of these sleep hygiene strategies may have some benefit, many of the self-management strategies reported by participants were unsuccessful, increasing feelings of helplessness, worry, frustration, and anger.[16] [Mixed population]
Anger	1	[16]	
Fear	1	[4]	– *"I wasn't even taking them for pain. I was just taking them to sleep. But then I immediately weaned myself off of them, because I was afraid of – I've got to find out what the underlying issue is instead of just taking a pill to mask it."[4]* [Testicular cancer]
Depression	1	[16]	– *"[…] you are not interested in anything really...it makes you a bit depressed."[16]* [Mixed population]
Guilt	1	[16]	– *"[…] massive guilt about feeling tired all of the time […]."[16]* [Mixed population]
Personality changes	1	[16]	– Author commentary: "I don't feel like myself": A significant change in personality […] frequently reported following the onset of sleep disturbance.[16] [Mixed population]

1.5.3. Activities of Daily Living (ADLs)

Table S8 provides an overview of how sleep impairment affected patient functioning in terms of their daily activities.

Table S8. Activities of daily living (ADL) impacts of sleep impairment within the qualitative literature

Impact	Number of sources	Sources	Supporting quote(s)
**Difficulty completing daily activities/tasks, due to:**			
Lack of motivation	2	[5, 16]	– Author commentary: Participants understood that their low energy levels directly impacted their ability to be productive and complete professional or personal work, often describing feeling "*unmotivated*."[16] [Mixed population]
Reduced daytime activity	1	[16]	– Author commentary: Furthermore, these symptoms would often lead to reduced daytime activity and a tendency to engage in behaviours such as daytime napping and increased caffeine intake to mitigate the effects of insomnia.[16][Mixed population]
Reduced productivity	1	[5]	– *"Lower productivity: "I do get up early in the morning, and if I don't sleep well, then I'm really sluggish and I'm not really that productive during the day."[5]* [Breast cancer]
**Affected activities:**			
General functioning /needs	8	[1, 7-10, 16]	– Author commentary: YWBC [young women with breast cancer] reported that sleep was "extremely important" to function.*[7]* [Breast cancer]<br>– *"It's seen as very important to manage sleep and its impacts on the quality of the rest of your life, because you're looking at basic needs, aren't you?"[10]* [Brain tumor]
Cleaning	1	[8]	– *"On top of that I just feel rubbish and I find it very difficult to do even you know clean the house be bothered to get out my pyjamas so it really does impact me. So yes, it's a bit of a battle. I generally just try and sort of forced my way through it and then maybe have a little nap."[8]* [Breast cancer]
Getting dressed	1		
Going for a walk	1	[9]	– *"Well, it affects me, because like yesterday, I was tired. We went to the shops for little of walk, I said [Caregiver*

Impact	Number of sources	Sources	Supporting quote(s)
			name] I can't. I'm way too tired, […]. So, we went home. If I had, if it wasn't something like that, I probably walked a bit longer."[9] [Brain tumor]
Needing to take naps	6	[7-9, 14-16]	– *"Not as bad as before, even if now I have busy day I get tired and so have to take a nap."[7]* [Breast cancer]<br>– *"I try and have a nap, well, I don't always have a nap, but I do get very tired in the afternoon and I'd say it's due to the chemo, because that makes you unwell. […] and it lingers afterwards as well like sleep wise. I just feel really um... tired a lot."[9]* [Brain tumor]<br>– *"You constantly want to take a nap."[15]* [testis carcinoma]

Abbreviations: ADL, activities of daily living; YWBC, Young women with breast cancer

1.5.4. Social Functioning and Relationships

Table S9 presents an overview of the social and relationship impacts associated with sleep impairment.

Table S9. Social and relationship impacts of sleep impairment within the qualitative literature

Impact	Number of sources	Sources	Supporting quote(s)
Relationships affected by irritability	2	[5, 8]	– Author commentary: In addition, some participants reported that a lack of restful sleep affected their daytime mood, including increased levels of daytime anxiety and irritability toward others. This, in turn, affected social functioning and relationships.[5] [Breast cancer]
Difficulty participating in social events	1	[16]	– *"I don't mix with people when I've had a bad night's sleep."[16]* [Mixed population]
Difficulty relating to other people			– Author commentary: These difficulties were compounded by a perception that significant others did not understand or empathize with such personality changes, and expected them to be the person they were prior to their cancer diagnosis.[16] [Mixed population]

Impact	Number of sources	Sources	Supporting quote(s)
Difficulty making social plans			– Author commentary: There was a tendency to no longer make plans or commit to anything due to the unpredictable nature of sleep and concern that they might be 'bad tempered' with others. One participant described it as being in 'fear of having to do something'.[16] [Mixed population]
Reduced quality time (due to sleeping separately)			– Author commentary: A number of participants reported sleeping in a different bedroom from their partner in order to avoid disturbing them during periods of night-time wakefulness. This enforced separation resulted in participants feeling that 'their relationship had changed' and that they 'missed out on conversations and making plans with their partners' at bedtime or the following morning.[16] [Mixed population]

1.5.5. Hobbies and Leisure Activities

Table S10 presents an overview of the impacts to leisure activities associated with sleep impairment.

Table S10. Leisure activity impacts of sleep impairment within the qualitative literature

Impact	Number of sources	Sources	Supporting quote(s)
Difficulty participating in interests	1	[5]	– Author commentary: One participant described how a lack of consistent sleep affected her motivation to engage in health behaviours (e.g., exercise), despite a desire to do so.[5] [Breast cancer]

1.5.6. Work and Financial

Table S11 presents an overview of the impacts of impaired sleep on work.

Table S11. Work impacts of sleep impairment within the qualitative literature

Impact	Number of sources	Sources	Supporting quote(s)
Difficulty completing work tasks	1	[1]	– Author commentary: Twelve patients reported fatigue, with lack of energy attributed to the disease itself, poor sleep, emotional exhaustion and side-effects of treatment. Lack of energy prevented

Impact	Number of sources	Sources	Supporting quote(s)
			patients from accomplishing tasks at work and at home.[1] [Lymphoma]
Need to reduce work hours	1	[8]	– Author commentary: […] negatively impacted their ability to work, as they struggled to wake up early and concentrate throughout the day. Some were even forced to cut down their working hours as a result.[8] [Breast cancer]
Unable to work	1	[16]	– Author commentary: […] decided to give up work due to the impact of insomnia on their daily functioning, with one describing it as being forced into retiring *"basically because I was so tired".[16]* [Mixed population]

1.5.7. Treatment

Table S12 presents an overview of the treatment impacts associated with sleep impairment.

Table S12. Treatment impacts of sleep impairment within the qualitative literature

Impact	Number of sources	Sources	Supporting quote(s)
Considering treatment discontinuation	1	[8]	– Author commentary: Participants felt that good quality sleep was essential for optimal daily functioning and when they did not achieve this, they were discouraged and frequently contemplated HT discontinuation.[8] [Breast cancer]

Abbreviations: HT, hormone therapy.

1.6. Coping Strategies

The coping strategies used by patients to manage impaired sleep are presented in Table S13.

Table S13. Coping strategies used to manage sleep impairment within the qualitative literature

Strategy	Number of sources	Sources	Supporting quote(s)
Using medications or supplements	10	[2, 4, 5, 7, 9, 10, 16-18, 22]	– Author commentary: Many spoke about a reluctance to take medication but felt like they did not have other options available.[4] [Mixed population]<br>– *"I've been to the doctor and he has given me, what's it called, melatonin, is it? […] which I think helps me fall asleep faster, but it doesn't seem to let me sleep all the way through. I've been unable to sleep all the way through now for some years."[9]* [Brain tumor]<br>– Author commentary: […] using herbal remedies to aid sleep.[16] [Mixed population]<br>– *"I was taking a sleeping pill every night, [zolpidem], to sleep. And if I just take like a half a* [medical cannabis] *gummy or something like that I can sleep and I wake up feeling very refreshed. And I sleep the entire night. With the [zolpidem], I still, sometimes, would wake up and when I'd wake up in the morning I would be very groggy for several hours because of that medicine."[22]* [Breast cancer]
Good sleep hygiene (maintaining sleep/wake schedule; having a nightly routine)	5	[4, 5, 7, 9, 16]	– *"I have a routine…routinized my sleep. Generally, go to bed at 10:30pm every night."[7]* [Breast cancer]<br>– *"Whatever you call your body rhythms all that […] you try to hopefully go to bed about the same time and get up about the same time so you're in the regular pattern."[9]* [Brain tumor]<br>– Author commentary: Other methods employed included an evening wind-down routine, sleep hygiene practices such as avoiding caffeine and chocolate later in the day […].[16] [Mixed population]
Avoiding/ reducing use of electronic devices	4	[4, 5, 7, 9]	– Author commentary: Some participants were aware of the connection between backlit devices and sleep disturbance and were actively trying to reduce their pre-bed screen time, acknowledging the negative impact of the device on sleep.[4] [Mixed population]<br>– *"It would be don't take computer bed, don't put the TV on when you go to bed, because it's stimulating*

Strategy	Number of sources	Sources	Supporting quote(s)
			my mind and I should be winding down. Sleep harm, that's what it's called."[9] [Brain tumor]
Calming/ relaxing activities	4	[7, 9, 10, 16]	– *"Sometimes I might do deep breathing. I'll relax."[7]* [Breast cancer]<br>– *"I just do like some breathing techniques to not worry about it."[9]* [Brain tumor]<br>– Author commentary: Wind-down routine.[16] [Mixed population]
Avoiding alcohol/ caffeine	4	[4, 7, 8, 16]	– Author commentary: Many participants reported an awareness of engaging in behaviours conducive with good sleep hygiene such as not having caffeine too late in the day.[4] [Mixed population]<br>– *"Caffeine—I don't drink coffee after 2-3pm in the afternoon."[7]* [Breast cancer]<br>– Author commentary: Some also limited caffeine intake during the afternoon, to prevent sleep disturbance.[8] [Breast cancer]
Managing environmental temperature	3	[5, 7, 8]	– *"I try to make sure that the house is cool so I can rest comfortably." "I try to have the house cool; very cool. Maybe around 66 [degrees], and that seems to help."[5]* [Breast cancer]<br>– *"In the winter, I keep the bedroom pretty cold but in summer, even with fan, cooling mattress and wicking clothes after all of that I still wake up 3-4 times a night."[7]* [Breast cancer]
Meditation/ Prayer	2	[5, 9]	– Author commentary: Some participants described prayer or meditation as a calming part of their bedtime routine that helped them fall asleep, including reading religious and motivational texts.[5] [Breast cancer]<br>– *"I believe in God, so I have faith and then I just say a few prayers and then hopefully fall asleep."[9]* [Brain tumor]
Changing medication timing/ routines	2	[8, 10]	– *"Things like reducing, giving dexamethasone in the morning and at lunchtime, […] we do, do that, but I don't know how effective it is at improving sleeping patterns, mainly because we never measure it."[10]* [Brain tumor]

Strategy	Number of sources	Sources	Supporting quote(s)
			– Author commentary: Most participants described the trial and error of implementing various routines, until a suitable one was established. This helped them to self-manage the impact of hormone therapy, by reducing the experience of certain side effects like nausea and poor sleep. While some preferred administering HT in the morning, others fared better by taking it at night."[8] [Breast cancer]
Staying active during daytime	2	[5, 9]	– *"Those are, up and down, tiring work… I was working in a preschool. So, you know I deal with the children a lot, back and forth on the playground. So, by the time you get home, and you do your little cooking and laundry and whatever, you're ready for bed around 9:30-10 pm. You get a good rest. And so, when I wake up, I'm fresh and busted with energy when I get up the next morning."[5]* [Breast cancer]
Psychological or behavioral interventions/ modifications (e.g., CBT)	2	[10, 17]	– Author commentary: Few HCPs described non-pharmacological options available; these included symptom education or introducing cognitive behavioural or meditative approaches to reduce anxiety and promote relaxation and mindfulness. Education (about fatigue, sleep and a healthy lifestyle, and practical steps such as increasing exercise) may have been delivered verbally or using written material during consultations. Other recommendations were support groups, psychoeducation about self-management for fatigue and psychosocial care for financial and social issues. However, resources varied greatly between institutions.[17] [Mixed population]
Managing environmental noise	1	[5]	– *"I just have to have something like that to make me fall asleep…Some background noise. No music or anything. Just something talking. And I have the radio right by my bed."* [Breast cancer]
Taking naps	1	[16]	– Author commentary: Daytime napping.[16] [Mixed population]
Avoiding social activities	1	[7]	– *"Even if I go out with friends-just hanging out in the evening, my mind feels very overstimulated."[7]* [Breast cancer]

Strategy	Number of sources	Sources	Supporting quote(s)
Getting up and doing something different/ thought distraction	1	[9]	– *"I think about things that I like to do. Like travelling or being able to get my driver's license back? Things like that."[9]* [Brain tumor]
Maintaining a positive outlook	1	[9]	– *"I think having a positive outlook to life—and even now I've still got a positive outlook, I've got no idea what my prognosis is."[9]* [Brain tumor]
Using caffeine	1	[16]	– Author commentary: Furthermore, these symptoms would often lead to reduced daytime activity and a tendency to engage in behaviors such as daytime napping and increased caffeine intake to mitigate the effects of insomnia.[16] [Mixed population]

Abbreviations: CBT, cognitive behavioral therapy.

1.7. References

[1] T. S. Bhat *et al.*, "Current measures are not sufficient: an interview-based qualitative assessment of quality of life in cutaneous T-cell lymphoma," *Br J Dermatol,* vol. 184, no. 2, pp. 310-318, Feb 2021, doi: 10.1111/bjd.19298.

[2] Y. C. Chang, G. M. Lin, T. A. Tseng, E. Vitale, C. H. Yang, and Y. L. Yang, "The Experience of Mindfulness-Based Stress Reduction on Menopausal Symptoms, Sleep Disturbance, and Body Image among Patients with Breast Cancer-A Qualitative Study," *Curr Oncol,* vol. 30, no. 1, pp. 1255-1266, Jan 16 2023, doi: 10.3390/curroncol30010097.

[3] R. Cheng, K. Scippa, F. L. Locke, J. T. Snider, and H. Jim, "Patient Perspectives on Health-Related Quality of Life in Diffuse Large B-Cell Lymphoma Treated with Car T-Cell Therapy: A Qualitative Study," *Oncol Ther,* vol. 10, no. 1, pp. 123-141, Jun 2022, doi: 10.1007/s40487-021-00174-0.

[4] S. N. Garland, F. K. Barg, B. Cakouros, P. Gehrman, K. N. DuHamel, and J. J. Mao, "A qualitative examination of the factors related to the development and maintenance of insomnia in cancer survivors," *Palliat Support Care,* vol. 17, no. 2, pp. 221-226, Apr 2019, doi: 10.1017/S1478951518000135.

[5] C. Geiss *et al.*, ""I Beat Cancer to Feel Sick:" Qualitative Experiences of Sleep Disturbance in Black Breast Cancer Survivors and Recommendations for Culturally Targeted Sleep Interventions," *Ann Behav Med,* vol. 56, no. 11, pp. 1110-1115, Nov 5 2022, doi: 10.1093/abm/kaac035.

[6] I. Guillemin, M. Darpelly, B. Wong, A. Ingelgard, and I. Griebsch, "Development of a disease conceptual model of patient experience with metastatic colorectal cancer: identification of the most salient symptoms and impacts," *J Cancer Surviv,* pp. 1-11, Dec 14 2022, doi: 10.1007/s11764-022-01279-9.

[7] Y. Hwang, S. Conley, N. S. Redeker, T. Sanft, and M. T. Knobf, "A qualitative study of sleep in young breast cancer survivors: "No longer able to sleep through the night"," *J Cancer Surviv,* Jan 21 2023, doi: 10.1007/s11764-023-01330-3.

[8] M. Ibrar, N. Peddie, S. Agnew, A. Diserholt, and L. Fleming, "Breast Cancer Survivors' Lived Experience of Adjuvant Hormone Therapy: A Thematic Analysis of Medication Side Effects and Their Impact on Adherence," *Front Psychol,* vol. 13, p. 861198, 2022, doi: 10.3389/fpsyg.2022.861198.

[9] M. S. Jeon, M. R. Agar, E. S. Koh, A. K. Nowak, E. J. Hovey, and H. M. Dhillon, "Understanding sleep disturbance in the context of malignant brain tumors: a qualitative analysis," *Neurooncol Pract,* vol. 8, no. 2, pp. 179-189, Apr 2021, doi: 10.1093/nop/npaa081.

[10] M. S. Jeon, M. R. Agar, E. S. Koh, A. K. Nowak, E. J. Hovey, and H. M. Dhillon, "Barriers to managing sleep disturbance in people with malignant brain tumours and their caregivers: a qualitative analysis of healthcare professionals' perception," *Support Care Cancer,* vol. 29, no. 7, pp. 3865-3876, Jul 2021, doi: 10.1007/s00520-020-05970-4.

[11] M. L. Martin, K. Halling, D. Eek, and M. Reaney, ""Lower abdominal pains, as if I was being squeezed...in a clamp": A Qualitative Analysis of Symptoms, Patient-Perceived Side Effects and Impacts of Ovarian Cancer," *Patient,* vol. 13, no. 2, pp. 189-200, Apr 2020, doi: 10.1007/s40271-019-00393-8.

[12] N. Nathwani *et al.*, "Patient perspectives on symptoms, health-related quality of life, and treatment experience associated with relapsed/refractory multiple myeloma," *Support Care Cancer,* vol. 30, no. 7, pp. 5859-5869, Jul 2022, doi: 10.1007/s00520-022-06979-7.

[13] K. Nishimura *et al.*, "Conceptual assessment of HRQOL among Japanese non-metastatic castration-resistant prostate cancer (nmCRPC) patients," *Cancer Med,* vol. 12, no. 2, pp. 1762-1778, Jan 2023, doi: 10.1002/cam4.4955.

[14] M. Parthipan *et al.*, "Symptom experiences of older adults during treatment for metastatic prostate cancer: A qualitative investigation," *J Geriatr Oncol,* vol. 14, no. 1, p. 101397, Jan 2023, doi: 10.1016/j.jgo.2022.10.012.

[15] F. Pompilus, A. Ciesluk, P. Marquis, I. Griebsch, and M. Voorhaar, "Understanding the Patient Experience in NUT Carcinoma: Qualitative Interviews with Patients and Caregivers to Develop a Conceptual Framework," *Oncol Ther,* vol. 9, no. 2, pp. 591-605, Dec 2021, doi: 10.1007/s40487-021-00166-0.

[16] P. Reynolds-Cowie and L. Fleming, "Living with persistent insomnia after cancer: A qualitative analysis of impact and management," *Br J Health Psychol,* vol. 26, no. 1, pp. 33-49, Feb 2021, doi: 10.1111/bjhp.12446.

[17] J. Savard, C. Filion, M. P. Gagnon, A. Caplette-Gingras, L. Belanger, and C. M. Morin, "Barriers and facilitators to implementing a stepped care cognitive-behavioral therapy for insomnia in cancer patients: a qualitative study," *Support Care Cancer,* vol. 30, no. 8, pp. 6689-6698, Aug 2022, doi: 10.1007/s00520-022-07094-3.

[18] D. van de Wal *et al.*, "A patient's perspective on the side effects of tyrosine kinase inhibitors in the treatment of advanced and metastatic gastrointestinal stromal tumors," *Future Oncol,* vol. 19, no. 4, pp. 299-314, Feb 2023, doi: 10.2217/fon-2022-0730.

[19] I. van Eck *et al.*, "Health-Related Quality of Life Issues Experienced by Thoracic and Breast Sarcoma Patients: A Rare and Understudied Group," *J Clin Med,* vol. 10, no. 22, Nov 16 2021, doi: 10.3390/jcm10225334.

[20] S. L. Walker, V. Vaughan Dickson, and P. Z. Cacchione, "A Pilot Mixed-Methods Study of Malignant Pleural Mesothelioma Symptoms," *Oncol Nurs Forum,* vol. 49, no. 6, pp. 615-623, Oct 20 2022, doi: 10.1188/22.ONF.615-623.

[21] Y. Yang, X. Chen, X. Pan, X. Tang, J. Fan, and Y. Li, "The unmet needs of patients in the early rehabilitation stage after lung cancer surgery: a qualitative study based on Maslow's hierarchy of needs theory," *Support Care Cancer,* vol. 31, no. 12, p. 677, Nov 7 2023, doi: 10.1007/s00520-023-08129-z.

[22] E. S. Zhou, M. M. Nayak, P. R. Chai, and I. M. Braun, "Cancer patient's attitudes of using medicinal cannabis for sleep," *J Psychosoc Oncol,* vol. 40, no. 3, pp. 397-403, 2022, doi: 10.1080/07347332.2021.1910396.
